# How Are Patented AI, Software and Robot Technologies Related to Wage Changes in the United States?

**DOI:** 10.3389/frai.2022.869282

**Published:** 2022-06-14

**Authors:** Frank M. Fossen, Daniel Samaan, Alina Sorgner

**Affiliations:** ^1^Department of Economics, University of Nevada, Reno, NV, United States; ^2^IZA, Bonn, Germany; ^3^ILO, Geneva, Switzerland; ^4^Department of Business Administration, John Cabot University, Rome, Italy; ^5^Kiel Institute for the World Economy, Kiel, Germany

**Keywords:** artificial intelligence, software, robots, wage dynamics, labor market

## Abstract

We analyze the relationships of three different types of patented technologies, namely artificial intelligence, software and industrial robots, with individual-level wage changes in the United States from 2011 to 2021. The aim of the study is to investigate if the availability of AI technologies is associated with increases or decreases in individual workers' wages and how this association compares to previous innovations related to software and industrial robots. Our analysis is based on available indicators extracted from the text of patents to measure the exposure of occupations to these three types of technologies. We combine data on individual wages for the United States with the new technology measures and regress individual annual wage changes on these measures controlling for a variety of other factors. Our results indicate that innovations in software and industrial robots are associated with wage decreases, possibly indicating a large displacement effect of these technologies on human labor. On the contrary, for innovations in AI, we find wage increases, which may indicate that productivity effects and effects coming from the creation of new human tasks are larger than displacement effects of AI. AI exposure is associated with positive wage changes in services, whereas exposure to robots is associated with negative wage changes in manufacturing. The relationship of the AI exposure measure with wage increases has become stronger in 2016–2021 in comparison to the 5 years before.

**JEL Classification:** J24, J31, O33.

## Introduction

Recent literature on technological change and its consequences for labor markets has raised concerns that advances in artificial intelligence (AI) may result in a massive replacement of human labor with capital (e.g., Autor, 2015; Acemoglu and Restrepo, 2018a,b, 2019; Bessen, 2019; Acemoglu et al., 2020). Frey and Osborne (2017) influenced this discussion significantly by predicting that technological possibilities of digital technologies allow for the displacement of a large share of U.S occupations in the near future. This debate can be placed in the larger historical context of how technological change alters the demand for human labor. Profit-maximizing entrepreneurs would utilize a new technology if it is economically viable and choose a new capital-labor ratio in their production decision. This may be associated with an adjustment of labor demand and with an increase or decrease of the wage. Whether this has been the case for AI technologies is an empirical question that we attempt to address in this paper. We are using a recent dataset on individual wages for the United States and combine it with a new measure for patented AI, software, and robot technologies as provided by Webb (2020).

As some economists have argued, AI is a general-purpose technology, which is neither linked to a certain type of physical device, nor to a specific application in a specific economic sector (e.g., Brynjolfsson and McAfee, 2014). Empirical measurement of the employability of AI throughout the economy, and of its current and future capabilities, is therefore not trivial. Presently, the most common approach in the literature is to directly compare currently existing human tasks carried out by labor with current or expected future capabilities of any AI-driven machine (see, e.g., Frey and Osborne, 2017; Brynjolfsson et al., 2018). Out of these considerations, a new important strand in the literature has arisen that aims at developing precise quantitative measures of different types of technologies' impacts on individual worker's tasks and occupations. This literature also aims at assessing the change in demand for certain types of work as evidenced by changes in employment and wages. In general, there seems to be agreement among researchers that an empirical estimation of the impact of AI on labor requires a metric that links the exposure of certain labor market variables such as human tasks, occupations or human skills to AI or other types of technologies.

As of today, there exist a variety of AI scores that all measure somewhat different aspects of “AI exposure” and therefore offer different economic interpretations. The four widely discussed measures of impacts of digital technologies are the occupational computerization probabilities by Frey and Osborne (2017), suitability of workers' tasks for machine learning (SML) by Brynjolfsson et al. (2018) as well as the within-occupation standard deviation of these SML scores, and AI Occupational Impact scores (AIOI) presented by Felten et al. (2019). Fossen and Sorgner (2022) use the four above measures to analyze heterogeneous effects of new digital technologies on individual-level wage and employment dynamics in the United States for 2011–2018. The authors employ data from the Current Population Survey (CPS) and its Annual Social and Economic Supplement (ASEC) to construct a panel. The results indicate that labor-displacing digital technologies (as captured by the computerization probabilities and the SML scores) are associated with slower wage growth and higher probabilities of switching one's occupation and becoming non-employed. In contrast, labor-reinstating digital technologies (as measured by the standard deviation of SML scores and AI occupational impact scores) improve individual labor market outcomes.^1^ Workers with high levels of formal education are most affected by the new generation of digital technologies. Thus, it has been shown that existing measures of AI exposure capture different effects of AI on occupations. It is therefore crucial to understand how various existing and new measures of digital technologies are associated with individual labor market outcomes, as this will shed light on the following two important questions: First, does the technology already have observable associations with changes in individual workers' wages or other labor market outcomes? And second, what is the direction of these relationships?

The aim of this paper is to investigate—closely following the methodology of Fossen and Sorgner (2022)—the associations between new measures of patented technologies proposed by Webb (2020) and individual wage dynamics in the United States. Webb (2020) constructs three different metrics from patent data to measure the occupational exposure to three types of technologies: AI, software, and industrial robots. While we are particularly interested in the metric related to AI exposure, we include all three metrics in our empirical analysis to allow for a comparison between three different types of patented technologies.

Our results show that occupational exposure to AI is associated with increasing wages, whereas exposure to software and robots is associated with decreasing wages. They further indicate that the positive relationship of AI exposure with wage growth became stronger in 2016–2021 than in 2011–2015 and that it is stronger in services than in manufacturing. In contrast, exposure to robots is associated with wage decreases in manufacturing and became somewhat weaker over time. The results are robust to excluding the years of the Covid-19 pandemic. Fossen and Sorgner (2019) distinguish between “destructive” digitalization, when digital technology is used or can be used to replace labor, and “transformative” digitalization, when digital devices bring about changes in the way human work is performed, potentially an augmentation of work, without leading to a replacement of the activity. As we discuss in more detail below, our findings suggest that AI exposure can be cautiously interpreted as transformative digitalization, whereas exposure to non-AI software and robots can be interpreted as destructive digitalization in the sense that these technologies decrease labor demand.

The remainder of the paper proceeds as follows. Section Conceptual Background provides the theoretical background and highlights the need for developing new measures of workers' exposure to various types of technology. Section Data describes the measures of exposure to AI, software, and industrial robots proposed in Webb (2020). Our empirical strategy and results are presented in sections Methods and Empirical results. Section Discussion discusses the results and provides concluding remarks.

## Conceptual Background

Acemoglu and Restrepo (2018a) propose a task-based framework (the “AR model”) in which new automation technologies lead to capital taking over tasks previously performed by human labor—if economically feasible. This displacement effect then results in a decrease in labor demand. The AR model implies several effects of automation that might countervail the displacement effect. These include, for instance, productivity effects that can increase the demand for tasks that cannot be automated (Autor and Dorn, 2013; Goos et al., 2014; Autor, 2015; Bessen, 2019); creation of new tasks for human workers; and an increase in the overall demand for human labor due to increases in capital accumulation.

Based on the AR model, we can distinguish a scale effect, triggered through higher productivity and the accumulation of capital, and a structural effect. The scale effect raises labor demand as such and can therefore lead to increases in employment or wages. The structural effect causes a re-allocation of tasks between humans and machines, whereby this re-allocation can result in a reduction of tasks for humans (displacement effect) or in an increase through new or altered tasks. Since occupations can be interpreted as bundles of tasks, the structural effect on occupations can be interpreted as being partly “transformative,” that is, an occupation is altered but does not necessarily become obsolete, and as “destructive,” i.e., an occupation is being partially destroyed by making some of the human tasks it consists of obsolete. Accordingly, Fossen and Sorgner (2019) distinguish transformative digitalization from destructive digitalization and categorize U.S. occupations along these lines. The resulting policy implications can be very different. A permanent or long-lasting destruction of a large number of occupations may justify entirely new economic policies, for example the institution of a universal basic income (UBI). If, instead, most occupations are transformed, then a strong focus needs to be put on training and re-skilling of the workforce.

An important implication from the AR model is that the labor market effects of technologies strongly depend on the type of technology and the purpose it was designed for. This makes it clear that there is a pronounced need for developing more precise measures of occupational exposure to different types of technologies and understanding how they are related to individual labor market outcomes. For instance, Fossen and Sorgner (2019) interpret the occupational computerization probabilities developed by Frey and Osborne (2017) as a measure of destructive digitalization and the AI occupational impact score introduced by Felten et al. (2019) as measure of transformative digitalization.^2^ Since most existing measures are only available for U.S. occupations, Carbonero et al. (2021) propose a novel approach that allows to translate existing technology exposure scores that were developed for U.S. occupations into scores for occupations in other countries, including developing countries, and illustrate the method for the cases of Lao PDR and Viet Nam. In Carbonero et al. (2021), the authors use the SML (“suitability for machine learning”) score developed by Brynjolfsson and Mitchell (2017) and Brynjolfsson et al. (2018) as a measure for destructive digitalization. The SML score is determined for work activities linked to U.S. occupations as reported in the O^*^NET database. The work activities, and hence the SML scores, can be aggregated on the occupational level. Carbonero et al. (2021) use the variance of the SML scores within an occupation as an indicator of transformative digitalization.

In sum, it is not trivial to measure the exposure of different occupations to new AI-based technologies empirically. Therefore, it is important to develop new measures of occupational AI exposure that can grasp various aspects of AI capabilities, and then to empirically relate them with individual labor market outcomes. This will help better understand the size and the direction of technology impacts on workers' jobs.

## Data

### Measures of Occupational Exposure to AI, Software, and Robots

Webb (2020) proposes a new approach to measure impacts of different types of digital technologies on occupations. In a nutshell, his method is based on the fact that patent data contain descriptions of the capabilities of the patented technologies. He links textual patent descriptions pertaining to a certain type of technology, such as AI, with the descriptions of tasks used in U.S. occupations from the O^*^NET database sponsored by the U.S. Department of Labor. O^*^NET provides for each existing occupation a list of tasks that are typically carried out by workers in this occupation, and it ranks the importance of each task. For example, “Document and maintain records of precision agriculture information” is one of the tasks that O^*^NET identifies for the occupation of agriculture technicians. To link the textual descriptions from O^*^NET with the description of an AI patent Webb extracts verb-noun pairs by means of a natural language processing algorithm and uses these verb-noun pairs to quantify the overlap between patents and tasks. In the previous example, such a pair would be “(maintain, records).” Basically, the algorithm would look for AI patents that are described by the same verb-noun pair. Each task is then assigned an exposure score that is based on the relative prevalence of the verb-noun pair in the total set of analyzed patents. Thus, the higher the task exposure score, the more patents were identified that describe a technology related to this task. To aggregate the task-level scores to the level of occupations, weights are used that are constructed as an average of the frequency, importance, and relevance of each task to the occupation, as specified in O^*^NET. The weights are scaled to sum to one. As source for patent information, Webb (2020) employs the Google Patents Public Data database. He does not impose a time restriction, but due to a strong increase in patents over time, few patents were filed before the 1990s. Most patents were filed in the 21st century, in particular in software and even more so in AI (Webb et al., 2018).

Webb (2020) constructs the exposure measure for three types of technologies: AI, software, and industrial robots. Hence, a distinction of AI from other digital technologies is possible through his method. Potentially, one can then disentangle heterogeneous effects of these technologies on wages. To restrict the set of patents to these three specific types of technologies, they had to be precisely defined. For example, only industrial robots that are used in the manufacturing sector are considered “robots” (according to the standardized definition, ISO 8373). Software are programs for which every action it performs has been specified in advance by a human, as opposed to AI, which is defined as all forms of machine learning algorithms, supervised learning and reinforcement learning algorithms.

According to Webb (2020), labor market effects of robots and software are very different from those of AI since the occupational exposure to AI concerns different socioeconomic groups. Using census samples for the United States for the years 1980–2010, he finds that a change from the 25th to the 75th percentile of exposure to robots is associated with a decline in within-industry employment shares of between 9 and 18% and a decline in wages of between 8 and 14%. Male workers with lower education and lower wages are more exposed to robots than others. The results for software indicate that middle-wage occupations are most exposed to software. The exposure to software is also less sharply decreasing with education in comparison with robots. The direction of the effects of software on aggregated employment and wages is similar to that of robots, but the effects are smaller in size. For example, moving from the 25th to the 75th percentile of exposure to software is associated with a decline in within-industry employment shares of between 7 and 11% and a decline in wages of between 2 and 6%. Hence, Webb's (2020) finding for robots and software point toward what we coined “destructive effects” of digitalization for the United States. Overall, the replacement effect appears to dominate over labor-reinstating effects when robots and software are employed.

One limitation of Webb's analysis is that the effects of AI cannot be determined with the dataset 1980–2010 because many of the significant technological advances in AI occurred more recently. Webb analyzes the tasks that are related to the capabilities of AI and the corresponding occupations and shows that low-wage occupations are potentially among the least, and high-wage occupations are among the most exposed occupations. Highly educated individuals are more likely to be exposed to AI. Interestingly, the opposite pattern is observed for the occupational exposure to robots and software.

Based on his findings, Webb (2020) makes the assumption that the relationship between AI exposure and changes in wages has the same negative, approximately linear relationship as the relationship that existed between exposure to software and robots and changes in wages. To determine the likely impact of AI on the wage distribution, Webb (2020) runs a simulation and finds that AI could possibly compress wages in the middle of the distribution but expand inequality at the top. In the following section, we introduce a different dataset with very recent data on wages and individual worker characteristics to empirically test the associations of the technologies with wage changes. While we are primarily interested in estimating the relationship of AI technology with individual wage changes, we also use the other two metrics for occupational exposure to software and industrial robots to allow for comparison between these different types of technology.

### Individual-Level Panel Data

To estimate associations of technology exposure with individual-level wage changes we use the Annual Social and Economic Supplement (ASEC) of the Current Population Survey (CPS), a representative survey of households in the United States provided by the Census Bureau. Given that most recent advances and the diffusion of AI technologies only occurred over the last few years, we concentrate in the main estimations on the period 2016–2021. In supplemental estimations we use the longer period 2011–2021 and subperiods. The ASEC is always conducted in March and contains information on labor income. We use the IPUMS-CPS database provided by Flood et al. (2017), who match consecutive individual-level observations to construct rotating panel data, allowing us to link the March ASEC of two subsequent years for most respondents. We calculate hourly wage changes between *t*-1 and *t* for each respondent using information about the income and hours worked in the previous calendar year.

We merge Webb's three measures of technology exposure of occupations (exposure to AI, exposure to software, and exposure to robots) with the individual's occupation in the initial year, *t*-1, using a crosswalk of occupational codes. Some of the occupations coded in the ASEC combine more than one occupation in the more detailed SOC codes used by Webb (2020). In these cases, we aggregate the exposure scores by using their mean values weighted by the number of employees in the respective occupations in the United States as provided by the Bureau of Labor Statistics (2018). We can merge the exposure scores to 435 occupations in the ASEC. We standardize the exposure scores to facilitate interpretation of the regression coefficients.

## Methods

In our econometric analysis we follow closely the approach proposed by Fossen and Sorgner (2022). We regress wage growth on the three Webb measures and control variables based on the sample of working individuals:


(1) $$\begin{array}{l} \ln\left(wage_{i,t}\right)-\ln\left(wage_{i,t-1}\right)={\delta^{\prime }}_{\text{ 1}}techexp_{j(i,t-1)}+\delta_{2}switch_{i,t}\\ \;+{\delta^{\prime }}_{\;3}techexp_{j(i,t-1)}\times switch_{i,t}\\ \;+\eta^{\prime }v_{i,t-1}+\xi^{\prime }w_{i,t}+{\omega^{\prime }}_{k}\;z_{j(i,t-1)}\\ \;+\theta_{year(t)}^{w}+\vartheta_{ind(i,t-1)}^{w}\\ \;+\mu_{j(i,t-1)}^{w}+\epsilon_{it}. \end{array}$$

The dependent variable is the relative change in hourly labor income of individual *i* between calendar years *t*-1 and *t* (log approximation). In year *t*-1 the individual worked in occupation *j(i,t-1)*. The three key explanatory variables summarized in the vector **techexp**_*j*(*i, t*−1)_ are the exposures of occupation *j* to patented AI, software, and robot technology using the measures developed by Webb (2020). We include the three exposure measures simultaneously in the regression such that the partial effect of each technology type is identified keeping the others constant. We also include a wide set of control variables that might affect individual wage growth.

The vector of coefficients **δ**_1_ captures the effects of different types of technologies (robots, software, AI) on wage growth for individuals who do not switch their occupation. *switch*_*i, t*_ is a dummy variable indicating whether a respondent *i* switched the main occupation between the years *t*-1 and *t*, identified by a change in the occupational code. The idea is that some individuals whose jobs are heavily affected by technologies could be able to switch to a different occupation, thereby preventing a possible wage decline. Interaction terms between this dummy variable and the three exposure measures (with coefficients **δ**_3_) capture how much the impacts of the technologies in the previous occupation on the individual's wage growth change in case of an occupational switch.

**v**_*i, t*−1_ is a vector of 10 splines of the initial individual wage (*wage*_*i, t*−1_) controlling for a potential general change in the income distribution. The vector also includes dummy variables indicating incorporated or unincorporated self-employment in *t*-1. The vector **w**_*i, t*_ contains further individual-level controls at time *t*: gender, age, age square, marital status, number of children in the household, ethnicity, highest educational attainment, residence in a metropolitan area, 8 dummies for the US Census regions, and a constant. We also include year dummies, $\theta_{year\left(t\right)}^{w}$, and 52 major industry dummies, $\vartheta_{ind\left(i,t-1\right)}^{w}$, to control for industry exposure to international trade in *t*-1. The occupational dummies $\mu_{j\left(i,t-1\right)}^{w}$ capture the 2-digit level of the occupation codes provided in *t*-1.

Additional occupation-level variables **z**_*j*(*i, t*−1)_ account for remaining variation within these 2-digit groups of occupations: the mean hourly wage rate, the self-employment rate, and the required degree of formal education at the entry level obtained from the Bureau of Labor Statistics (2018); the share of female workers in each occupation computed directly from our microdata; the measure of susceptibility of occupations for offshoring provided by Blinder and Krueger (2013); and the occupations' routine manual and routine cognitive task intensities that we create from O^*^NET following Acemoglu and Autor (2011). We cluster standard errors at the level of occupations.

## Empirical Results

### All Workers

Table 1 shows sample means, standard deviations and correlation coefficients for the variables used in this analysis. Exposure to AI is positively correlated with software but less so with industrial robots. The correlations highlight the importance of including these technologies jointly in the regressions to estimate partial effects of each technology keeping the others constant. The raw correlation of wage growth with the AI exposure score is weakly negative and those with software and robot exposure are weakly positive (significant at the 5% level). As we will see below, these signs change in the multivariate regressions controlling for essential factors influencing wages at the individual and occupation levels. Exposure to robots is positively correlated with routine manual task intensity, confirming our expectations.

**Table 1:** Descriptive statistics.

	**Means**	**Std. dev**.	**Correlation coefficients**		
			**Exposure to AI**	**Exposure to software**	**Exposure to robots**
**Technology exposure measures**
Exposure to AI	0.379	0.217	1.000		
Exposure to software	0.421	0.245	0.532	1.000	
Exposure to robots	0.498	0.629	0.026	0.503	1.000
**Individual-level characteristics**
Annual wage growth	0.028	0.891	−0.011	0.018	0.022
Occupation switch	0.583		0.008	0.020	−0.027
Less than high school	0.063		−0.036	0.099	0.230
High school degree	0.271		−0.047	0.134	0.222
Some college	0.291		−0.030	0.022	−0.005
College degree	0.374		0.089	−0.193	−0.315
Male	0.514		0.169	0.134	0.187
Age	43.84	11.90	0.004	−0.038	−0.003
Metropolitan area	0.823		0.010	−0.047	−0.080
Married	0.620		0.045	−0.046	−0.067
Number of children in household	0.919		0.016	−0.011	0.005
White	0.824		0.022	−0.009	−0.027
Black	0.089		−0.026	0.021	0.052
Asian	0.058		0.007	−0.019	−0.037
Other race	0.029		−0.015	0.009	0.024
Self–employed (incorporated)	0.044		0.007	−0.059	−0.067
Self–employed (unincorporated)	0.057		−0.009	−0.018	0.012
**Occupation–level characteristics**
Mean hourly wage in occupation	29.72	18.68	0.177	−0.224	−0.382
Share of women in occupation	0.486	0.295	−0.287	−0.227	−0.316
Self-employment rate in occ.	0.102	0.150	0.010	−0.119	−0.129
Offshorability score in occ.	1.810	1.317	0.271	0.071	−0.218
Routine cognitive task intensity in occ.	0.034	0.973	−0.034	−0.199	−0.414
Routine manual task intensity in occ.	−0.206	0.817	−0.005	0.369	0.503
High school diploma needed	0.358		0.032	0.013	−0.121
Postsecondary non-degree needed	0.077		−0.106	0.027	0.106
Some college needed	0.020		−0.037	0.019	−0.050
Associate's degree needed	0.027		0.071	0.006	−0.049
Bachelor's degree needed	0.260		0.253	−0.131	−0.317
Master's degree needed	0.023		0.017	−0.061	−0.089
Doctoral or prof. degree needed	0.043		−0.072	−0.140	−0.133

*The table shows mean values, standard deviations for non-binary variables, and correlation coefficients. The exposure scores to AI, software and robots are not standardized here. Number of person-month observations: 58,394*.

*Source: Own calculations based on the ASEC 2016-21*.

We present the main estimations (Equation 1) of the controlled associations of Webb's three different technology exposure measures with wage growth in Table 2 based on the period 2016–2021. The four models include different sets of control variables whereby the preferred model (1) contains all variables discussed in the previous section with industry group, occupation group and time dummies, as well as all occupation-level controls. In models (2) – (4), the occupational variables and the occupation- and industry fixed effects are successively excluded from the estimation as robustness checks. These estimations are based on larger samples because they include observations with missing values in the occupation-level or industry variables.

**Table 2:** Relationship of technology exposure with annual wage growth (2016–2021).

	**(1)**	**(2)**	**(3)**	**(4)**
Occupation switch	−0.0393***	−0.0431***	−0.0459***	−0.0408***
	(0.0112)	(0.00966)	(0.0112)	(0.0144)
Exposure to AI	0.0268**	0.0501***	0.0595***	0.0796***
	(0.0115)	(0.0132)	(0.0136)	(0.0167)
Exposure to AI *x* occupation switch	−0.0371***	−0.0402***	−0.0353***	−0.0419***
	(0.0117)	(0.0116)	(0.0122)	(0.0138)
Exposure to software	−0.0326**	−0.0508**	−0.0531***	−0.0508**
	(0.0162)	(0.0201)	(0.0177)	(0.0230)
Exposure to software *x* occupation switch	0.0404**	0.0388**	0.0408**	0.0438**
	(0.0162)	(0.0167)	(0.0170)	(0.0193)
Exposure to robots	−0.0246**	−0.0307**	−0.0493***	−0.0542***
	(0.0115)	(0.0143)	(0.0125)	(0.0142)
Exposure to robots *x* occupation switch	0.0299**	0.0249**	0.0246*	0.0250*
	(0.0132)	(0.0122)	(0.0131)	(0.0134)
High school degree	0.0804***	0.0934***	0.102***	0.113***
	(0.0141)	(0.0141)	(0.0138)	(0.0150)
Some college	0.139***	0.156***	0.173***	0.181***
	(0.0153)	(0.0152)	(0.0156)	(0.0178)
College degree	0.296***	0.327***	0.373***	0.375***
	(0.0177)	(0.0173)	(0.0181)	(0.0215)
Male	0.146***	0.146***	0.154***	0.168***
	(0.00716)	(0.00720)	(0.00751)	(0.0101)
Age	0.0283***	0.0308***	0.0311***	0.0345***
	(0.00257)	(0.00233)	(0.00234)	(0.00300)
Age squared	−0.000295***	−0.000323***	−0.000328***	−0.000366***
	(0.0000298)	(0.0000271)	(0.0000271)	(0.0000356)
Marital status	0.0768***	0.0728***	0.0778***	0.0832***
	(0.00940)	(0.00870)	(0.00869)	(0.00881)
Number of children in household	0.00276	0.00106	−0.000225	−0.00269
	(0.00296)	(0.00286)	(0.00284)	(0.00303)
Metropolitan area	0.0740***	0.0753***	0.0754***	0.0745***
	(0.00882)	(0.00786)	(0.00809)	(0.00845)
Black	−0.0689***	−0.0744***	−0.0816***	−0.0759***
	(0.0131)	(0.0118)	(0.0117)	(0.0116)
Asian	−0.0254*	−0.0242*	−0.0235	−0.0190
	(0.0149)	(0.0142)	(0.0154)	(0.0171)
Other race	−0.0536**	−0.0371*	−0.0367*	−0.0423**
	(0.0208)	(0.0205)	(0.0206)	(0.0205)
Self-employed (unincorporated)	−0.157***	−0.168***	−0.171***	−0.172***
	(0.0275)	(0.0243)	(0.0227)	(0.0255)
Self-employed (incorporated)	−0.0418**	−0.0484**	−0.0326*	−0.0448**
	(0.0210)	(0.0189)	(0.0176)	(0.0203)
Hourly wage in occupation	0.00276***			
	(0.000778)			
Share of women in occupation	−0.0702**			
	(0.0330)			
Self–employment rate in occupation	−0.109**			
	(0.0503)			
High school needed	0.0935***			
	(0.0214)			
Post–secondary degree needed	0.0455			
	(0.0279)			
Some college needed	0.0488			
	(0.0349)			
Associated degree needed	0.0413			
	(0.0425)			
Bachelor degree needed	0.104***			
	(0.0325)			
Master degree needed	0.179***			
	(0.0453)			
Doc. or professional degree needed	0.214***			
	(0.0470)			
Offshoreability score in occupation	−0.00197			
	(0.00669)			
Routine cognitive task intensity	0.0266***			
	(0.00732)			
Routine manual task intensity	−0.0252**			
	(0.0101)			
Constant	1.305***			
	(0.0918)			
Further individual controls, income splines	Yes	Yes	Yes	Yes
Year dummies	Yes	Yes	Yes	Yes
Industry dummies	Yes	Yes	Yes	–
Occupation dummies (2 digits)	Yes	Yes	–	–
Occupation–level controls	Yes	–	–	–
Number of observations	58,394	69,434	69,434	70,650
R^2^	0.304	0.297	0.288	0.274

OLS regressions. The dependent variable is the growth rate in the hourly wage between two adjacent years in real US$ (logarithmic approximation). The exposure measures pertain to the first year of a 2-year pair. The switch dummy variable indicates that an individual switched to a new occupation between the 2 years. We interact this dummy variable with the exposure measures. The standard errors are clustered at the level of occupations. Stars (***/**/*) indicate significance at the 1/5/10% level.

*Source: Own calculations based on the ASEC 2016-21*.

We are mainly interested in the coefficients of the three technology exposure measures, i.e., exposure to AI, exposure to software, and exposure to industrial robots. We can see that the estimates of the coefficients are consistent in terms of signs and are significant at the 1 or 5 percent levels in all four models, indicating robustness of our estimation.

Exposure to software and robots is associated with a decrease in the growth rate of individual hourly labor income. In model (1) with full controls, a one standard deviation higher exposure to software is associated with a 3.26 percentage points lower annual wage growth, and a one standard deviation higher exposure to robots is related to a 2.46 percentage points lower annual wage growth. On the contrary, we find a *positive* association of wage growth with exposure to AI. We find a 2.68 percentage points higher wage growth for a one standard deviation increase in exposure to AI. The coefficients of the interaction terms with occupation switch are significantly different from zero for the three technologies and always have the opposite sign from the coefficient of technology exposure. Thus, by switching occupation, an individual mitigates or even overcompensates the effect the technology exposure in the original occupation has on the wage.

Did the strengths of the associations change over time? In Table 3, we repeat the estimation of the full model but using different time periods^3^. The first column shows the estimates for the prolonged period of 2011–2021 and the second for the first 5 years (2011–2015); these results can be compared to the main estimation using the last 5 years (2016–2021) in model (1) in Table 2. We can see that the positive association of AI exposure with wage changes is stronger in 2016–2021 than in the 5 years before. This observation might reflect that the diffusion of AI technologies has accelerated in the last 5 years. The relationship of exposure to software with wage dynamics remained unchanged over these 10 years while that of exposure to robots became somewhat weaker.

**Table 3:** Technology exposure and annual wage growth in different periods.

	**2011–2021**	**2011–2015**	**2016–2019**
Occupation switch	−0.0396***	−0.0387***	−0.0366***
	(0.00955)	(0.0101)	(0.0116)
Exposure to AI	0.0214**	0.0166**	0.0271**
	(0.00866)	(0.00835)	(0.0123)
Exposure to AI *x* occupation switch	−0.0331***	−0.0292***	−0.0332***
	(0.00905)	(0.00915)	(0.0125)
Exposure to software	−0.0331***	−0.0324***	−0.0376**
	(0.0119)	(0.0100)	(0.0179)
Exposure to software *x* occupation switch	0.0346***	0.0300***	0.0421**
	(0.0117)	(0.0108)	(0.0186)
Exposure to robots	−0.0278***	−0.0289***	−0.0259**
	(0.00983)	(0.0105)	(0.0115)
Exposure to robots *x* occupation switch	0.0312***	0.0311***	0.0339***
	(0.0111)	(0.0113)	(0.0128)
Further individual controls, income splines	Yes	Yes	Yes
Year dummies	Yes	Yes	Yes
Industry dummies	Yes	Yes	Yes
Occupation dummies (2 digits)	Yes	Yes	Yes
Occupation–level controls	Yes	Yes	Yes
Number of observations	131,539	73,145	50,385
*R* ^2^	0.306	0.320	0.307

OLS regressions for different periods. The dependent variable is the growth rate in the hourly wage between two adjacent years in real US$ (logarithmic approximation). The exposure measures pertain to the first year of a two–year pair. The switch dummy variable indicates that an individual switched to a new occupation between the 2 years. We interact this dummy variable with the exposure measures. All control variables listed in model (1) of Table 2 are included in the regressions but not shown. The standard errors are clustered at the level of occupations. Stars (***/**/*) indicate significance at the 1/5/10% level.

*Source: Own calculations based on the ASEC 2011-21*.

One might wonder if the results are driven by the Covid-19 pandemic, which changed work in dramatic ways including widespread shifts to remote work from home. To assess the sensitivity of our results, in the rightmost column of Table 3 we exclude the years of the pandemic, 2020 and 2021, from our main sample, thus leaving the period 2016–2019^4^. The results are very similar to those in model (1) in Table 2, so we conclude that our findings are not driven by the COVID-19 pandemic.

### Different Groups of Workers

Are occupational exposures to the technologies associated with stronger wage changes for certain groups of workers? In this section we split the sample by worker characteristics and run our preferred regression with the full set of controls, similar to model (1) in Table 2. We use the sector and type of worker in the initial year, *t*-1, to split the samples. Results in Table 4 show that the relationships of exposure to AI and software with wage changes are strongest in the services sector, while the point estimates are insignificant in the manufacturing sector^5^. In contrast, exposure to robots is more strongly related to decreasing wages in manufacturing and unrelated to wage change in services. These links between the different technologies and sectors are consistent with expectations given the nature and use of the technologies, for example, the deployment of industrial robots in manufacturing, and underline the plausibility of our results. Moreover, employee's wage dynamics are mostly related to AI technologies and robots, while earnings of entrepreneurs are significantly associated only with software exposure. A possible explanation for this latter result could be that software may perform tasks that firms have previously subcontracted to entrepreneurs.

**Table 4:** Technology exposure and annual wage growth by sector and employment status.

	**Services**	**Manufacturing**	**Employees**	**Entrepreneurs**
Occupation switch	−0.0312**	−0.0796***	−0.0449***	0.0893**
	(0.0126)	(0.0212)	(0.0108)	(0.0413)
Exposure to AI	0.0234*	0.0214	0.0247**	0.0626
	(0.0133)	(0.0172)	(0.0105)	(0.0467)
Exposure to AI *x* occupation switch	−0.0372***	−0.0274	−0.0417***	−0.0308
	(0.0140)	(0.0209)	(0.0113)	(0.0523)
Exposure to software	−0.0336**	−0.0266	−0.0296*	−0.171***
	(0.0167)	(0.0238)	(0.0156)	(0.0636)
Exposure to software *x* occupation switch	0.0387**	0.0427	0.0413***	0.138*
	(0.0180)	(0.0291)	(0.0154)	(0.0772)
Exposure to robots	−0.0174	−0.0601***	−0.0235**	0.000745
	(0.0122)	(0.0163)	(0.0117)	(0.0484)
Exposure to robots *x* occupation switch	0.0280*	0.0323	0.0221*	0.0884
	(0.0158)	(0.0200)	(0.0120)	(0.0601)
Further individual controls, income splines	Yes	Yes	Yes	Yes
Year dummies	Yes	Yes	Yes	Yes
Industry dummies	Yes	Yes	Yes	Yes
Occupation dummies (2 digits)	Yes	Yes	Yes	Yes
Occupation–level controls	Yes	Yes	Yes	Yes
Number of observations	46,265	11,425	52,494	5,900
R^2^	0.307	0.332	0.319	0.350

OLS regressions for different sectors and by employment status. The dependent variable is the growth rate in the hourly wage between two adjacent years in real US$ (logarithmic approximation). The exposure measures pertain to the first year of a two-year pair. The switch dummy variable indicates that an individual switched to a new occupation between the 2 years. We interact this dummy variable with the exposure measures. All control variables listed in model (1) of Table 2 are included in the regressions but not shown. The standard errors are clustered at the level of occupations. Stars (***/**/*) indicate significance at the 1/5/10% level.

*Source: Own calculations based on the ASEC 2016-21*.

In Table 5 we split the sample by demographic characteristics. Occupational exposure to robots is negatively related with wage growth of both male and female workers, although it is only statistically significant for males. The effects of AI and software exposure are comparable for both genders. Moreover, the associations of technology exposure with wage changes are statistically significant only for workers residing outside the core cities in the United States. The point estimates have the same signs within core cities, however, and the statistical insignificance there may be due to the smaller sample size of core city residents.

**Table 5:** Technology exposure and annual wage growth by demographics.

	**Female**	**Male**	**Core city**	**Other areas**
Occupation switch	−0.0414***	−0.0361**	−0.0566***	−0.0372***
	(0.0151)	(0.0143)	(0.0154)	(0.0126)
Exposure to AI	0.0283**	0.0319***	0.0130	0.0293**
	(0.0143)	(0.0121)	(0.0150)	(0.0133)
Exposure to AI *x* occupation switch	−0.0407***	−0.0375***	−0.0312*	−0.0366***
	(0.0157)	(0.0131)	(0.0163)	(0.0132)
Exposure to software	−0.0345*	−0.0277*	−0.0281	−0.0322*
	(0.0178)	(0.0161)	(0.0180)	(0.0187)
Exposure to software *x* occupation switch	0.0419**	0.0412***	0.0277	0.0443**
	(0.0199)	(0.0157)	(0.0201)	(0.0179)
Exposure to robots	−0.0231	−0.0341***	−0.0313	−0.0229*
	(0.0161)	(0.0128)	(0.0211)	(0.0131)
Exposure to robots *x* occupation switch	0.0351*	0.0267**	0.0228	0.0354***
	(0.0180)	(0.0133)	(0.0240)	(0.0116)
Further individual controls, income splines	Yes	Yes	Yes	Yes
Year dummies	Yes	Yes	Yes	Yes
Industry dummies	Yes	Yes	Yes	Yes
Occupation dummies (2 digits)	Yes	Yes	Yes	Yes
Occupation–level controls	Yes	Yes	Yes	Yes
Number of observations	28,358	30,036	14,530	34,353
R^2^	0.334	0.293	0.310	0.303

OLS regressions by gender and in core cities vs. other areas. The dependent variable is the growth rate in the hourly wage between two adjacent years in real US$ (logarithmic approximation). The exposure measures pertain to the first year of a two–year pair. The switch dummy variable indicates that an individual switched to a new occupation between the 2 years. We interact this dummy variable with the exposure measures. All control variables listed in model (1) of Table 2 are included in the regressions but not shown. The standard errors are clustered at the level of occupations. Stars (***/**/*) indicate significance at the 1/5/10% level.

*Source: Own calculations based on the ASEC 2016-21*.

## Discussion

The aim of this paper is to investigate the relationships of three types of patented technologies, AI, software and industrial robots, with individual wage dynamics in the United States. To this end, we employ three measures of occupational exposure to these technologies developed by Webb (2020) that he constructs based on the textual descriptions of patents and of tasks that workers perform in their occupations. While Webb (2020) provides empirical evidence for how his measures of exposure to software and robots are associated with employment and wage dynamics at the level of occupations and industries during 1980–2010, we add to this evidence by focusing on the micro-level of individual workers in a more recent period from 2016 to 2021. Importantly, using these recent data allows us to also estimate associations between wage changes and exposure of occupations to AI since the dissemination and implementation of AI technologies has accelerated considerably.

In a nutshell, we find, consistently with the AR model and previous empirical literature, that different types of technology are related to labor markets in different ways. Industrial robots and software are associated with decreasing individual wages, although the relationship has become weaker for robots in more recent years. In contrast, occupational exposure to AI technologies, which are defined as machine learning algorithms, supervised learning and reinforcement learning algorithms, is associated with a positive individual wage growth, controlling for other relevant factors. Remarkably, the strength of the relationship of AI exposure with wage growth has increased over the last decade, which may indicate that firms have started to implement these technologies at a larger scale. AI exposure is associated with wage dynamics in services and robots exposure with wage dynamics in manufacturing. Wages of individuals who switch their occupations are not affected by the exposure of their initial occupation to these technologies.

The opposite signs of the relationships with wage growth show that exposure to AI is very different from exposure to software and robots. Our results are consistent with the interpretation that software and robots entail a much stronger displacement effect on workers and hence exhibit destructive forms of digitalization, whereas AI rather transforms occupations and may make human workers more productive.

Our estimation results for 2016–2021 contrast with the simulation results by Webb (2020). By assuming that the relationship of wage changes with AI exposure will be the same negative relationship as it was with exposure to robots and software in the past, he predicts that AI exposure will decrease wages at the 90th percentile relative to the 10th percentile in the future. However, our estimation results suggest that this assumption is questionable because we find that, contrary to robots and software, the association between wage changes and AI exposure is positive.

When comparing Webb's AI exposure measure to other available measures of AI, the positive relationship with wage changes in recent US data is similar to results reported by Fossen and Sorgner (2022) for Felten et al.'s (2019) AI Occupational Impact scores, which reflect past progress in AI fields and therefore are likely to capture the transformative effects of AI on work. However, the positive relationship of Webb's AI measure contrasts with Brynjolfsson et al.'s (2018) measure of suitability of tasks for machine learning that was found to be negatively associated with individual wage growth in the US (Fossen and Sorgner, 2022), thus, indicating a destructive nature of this particular subfield of AI technologies. While Webb's measure includes machine learning technologies, it remains unclear why the effect of this measure is contrary to the one by Brynjolfsson et al. (2018). A possible explanation could be that other subfields of AI that lead to productivity effects or create new tasks for human workers may overweigh the negative effect coming from ML technologies. If so, this calls for the development of new, more fine-grained measures of occupational exposure to various subfields of AI.

Our study is not without limitations. For instance, it is unclear to what extent the AI technologies reflected in the patents were already implemented in occupations in our estimation period 2016–2021. When these AI technologies will be more fully implemented in the future, the relationship with wage changes may change its direction, even though our findings for this rather early stage in this technology's lifecycle suggest otherwise. Moreover, we were not able to establish a causal relationship between the technology exposure scores and wage dynamics. Future research should try to find ways to estimate the causal impact of AI technologies on workers' economic outcomes. Another interesting question for future research would be to investigate how individuals use various risk mitigation strategies to deal with negative impacts of technologies on their jobs. Our study indicates that occupational switching is a potential route to minimize such risks, but it is certainly costly and not equally available to all affected workers. Last but not least, the Covid-19 pandemic has accelerated digital transformation processes, which might soon become observable in changing individual economic outcomes. Thus, future research could investigate the impact of the surge in digitalization of work processes on individual workers when these data become available.

In conclusion, exposure of occupations to patented AI technologies is positively associated with individual wage growth, as opposed to patented software and robot technologies. More research is needed on developing precise measures of specific AI technology impacts on workers' jobs and on assessing the labor market consequences.

## Data Availability Statement

Publicly available datasets were analyzed in this study. This data can be found at: https://cps.ipums.org/cps/ and https://www.michaelwebb.co/.

## Author Contributions

All authors listed have made a substantial, direct, and intellectual contribution to the work and approved it for publication.

## Conflict of Interest

The authors declare that the research was conducted in the absence of any commercial or financial relationships that could be construed as a potential conflict of interest.

## Publisher's Note

All claims expressed in this article are solely those of the authors and do not necessarily represent those of their affiliated organizations, or those of the publisher, the editors and the reviewers. Any product that may be evaluated in this article, or claim that may be made by its manufacturer, is not guaranteed or endorsed by the publisher.

## Footnotes

^1^Classification of these AI impact measures in terms of labor-displacing vs. labor-reinstating effects is based on empirical associations between these measures with individual labor market outcomes, as discussed in Fossen and Sorgner (2022). The authors of the measures themselves do not provide such a qualitative assessment for their impact scores.

^2^Please note that this classification is based on empirical insights and is not defined ex ante by the authors who developed them.

^3^The full set of control variables is included in all regressions but the estimates are not shown in the table.

^4^On March 11, 2020, the World Health Organization declared COVID-19 a pandemic. On March 16, the San Francisco Bay Area imposed the first shelter-in-place restrictions in the United States followed by the State of California on March 19 and New York State the next day. Thus, the March 2020 ASEC collection is likely to reflect the early impacts of the pandemic, and the March 2021 ASEC was collected in the middle of the pandemic; on March 19, 2021, the first 100 million Covid vaccine doses were administered in the United States.

^5^In the column labeled “Manufacturing” we combine the primary and secondary sectors, but the agricultural sector accounts for only a very small employment share in Germany.
